# Short-term gains, long-term pains: a comparative study of percutaneous vertebroplasty vs. kyphoplasty in osteoporotic vertebral compression fracture

**DOI:** 10.3389/fendo.2026.1730448

**Published:** 2026-02-24

**Authors:** Chongyue Sun, Zhiling Qing, Shuang Xu, Qing Wang, Shuai Zhang

**Affiliations:** 1 Southwest Medical University, Luzhou, Sichuan, China; 2 Department of Radiology, Kandze Prefecture People’s Hospital, Kangding, Sichuan, China; 3 Department of Orthopedics, The Affiliated Hospital of Southwest Medical University, Southwest Medical University, Luzhou, Sichuan, China

**Keywords:** bone cement distribution, deformity correction, osteoporotic vertebral compression fracture, percutaneous kyphoplasty, percutaneous vertebroplasty

## Abstract

**Objective:**

To compare the clinical efficacy and long-term deformity correction retention of percutaneous vertebroplasty (PVP) and percutaneous kyphoplasty (PKP) in the treatment of osteoporotic vertebral compression fractures (OVCF).

**Methods:**

We retrospectively analyzed clinical data from patients with OVCF admitted to our hospital between January 2020 and January 2023. Patients were allocated to either the PVP or PKP group. Baseline characteristics, intraoperative variables, deformity correction [anterior vertebral height (AVH) and local Cobb angle (CA)], and symptom relief [visual analog scale (VAS) scores for back pain] were compared. The incidence of bone cement leakage was assessed, and bone cement distribution patterns were compared between the procedures. In the PKP group, the correlation between cement distribution pattern and both deformity correction loss and symptom improvement were also analyzed.

**Results:**

Postoperatively, PKP demonstrated superior immediate anatomical restoration with greater AVH improvement (4.02 ± 0.34 mm vs. PVP 2.44 ± 0.21 mm, p = 0.005) and CA correction (-4.67° ± 0.32° vs. -3.02° ± 0.17°, p = 0.007). Both groups achieved comparable substantial symptom relief: median Oswestry Disability Index (ODI) decreased by 39.8 points in PVP and 39.7 points in PKP (p = 0.107), and median VAS decreased by 6.0 points in both groups (p = 0.420). However, at 2-year follow-up, PKP exhibited significant reversal: greater AVH correction loss (7.50% ± 0.97% vs. PVP 1.45% ± 0.57%, p < 0.001), CA correction loss (7.64% ± 1.12% vs. 1.10% ± 0.58%, p < 0.001), and worsened symptoms (VAS median 3.0 vs. 2.0, p =0.014; ODI 28.8% vs. 21.5%, p < 0.001). Critical to this divergence, PVP achieved superior cement distribution—reflected in higher Cement Distribution Score (CDS) (10.0 [IQR 9–10] vs. PKP 8.0 [8–9], p < 0.001) and Specific Surface Area (SSA) (5.66 ± 0.61 vs. 4.41 ± 0.67 cm²/cm³, p < 0.001)—with both parameters negatively correlating with loss of both AVH and CA correction, while positively correlating with improvement rates in VAS and ODI scores (all p < 0.001). PVP was associated with a significantly higher incidence of bone cement leakage compared to PKP (19.8% [20/101] vs. 8.42% [8/95], p = 0.024). PKP also incurred higher adjacent vertebral fractures (9.45% [9/95] vs. 1.98% [2/101], p = 0.023), augmented vertebra refractures (8.42% [8/95] vs. 1.98% [2/101], p = 0.041), longer operative time (44.15 ± 10.09 vs. 40.62 ± 11.71 min, p = 0.019), increased radiation exposure (24.26 ± 2.90 vs. 21.80 ± 3.35 exposures, p < 0.001), and doubled cost (8042 ± 1608 vs. 4316 ± 863 USD, p < 0.001).

**Conclusion:**

Both PVP and PKP are effective short-term treatments for OVCF. Therefore, PVP may be superior to PKP in maintaining long-term correction, particularly when bone cement distribution is optimized.

## 1 Introduction

With the accelerating pace of population aging, osteoporosis has been identified by the World Health Organization (WHO) as one of the 10 most common chronic conditions posing a major threat to the health of older adults (1, 2). Among its complications, osteoporotic vertebral compression fracture (OVCF) is the most frequent, affecting nearly 40.0% of postmenopausal women with at least one fragility fracture over their lifetime, thereby creating a significant burden on families and society (3, 4).

OVCF often leads to debilitating thoracic or lumbar back pain, progressive spinal kyphotic deformity, and, in advanced cases, impairment of cardiopulmonary and gastrointestinal functions, resulting in a marked decline in patients’ quality of life (5, 6). Although some individuals experience relief through conservative approaches—including bed rest, oral non-steroidal anti-inflammatory drugs, brace support, and anti-osteoporotic therapy—more than one-third eventually require surgical treatment (7, 8). Owing to the advanced age, complex comorbidities, and severe bone loss in these patients, outcomes following traditional open reduction and internal fixation are often unsatisfactory (9, 10).

In 1987, Galibert first introduced bone cement injection into the vertebral body for treating vertebral hemangiomas, yielding favorable clinical outcomes (11). Percutaneous vertebroplasty (PVP), developed subsequently, stabilizes vertebral fractures by injecting bone cement, which rapidly restores spinal integrity and provides substantial relief of thoracolumbar pain (12, 13). However, PVP shows limited ability to restore vertebral height and is often inadequate in correcting kyphotic deformities (13, 14). Furthermore, the use of high-pressure cement injection is associated with a high incidence of cement leakage (15, 16). To overcome these limitations, Reiley et al. and Belkoff et al. proposed a balloon-assisted technique to expand the compressed vertebral body and create a cavity for cement injection (17). This method improves the correction of spinal kyphosis and, by compressing surrounding trabecular bone, helps seal fracture lines, thereby significantly lowering the risk of cement leakage (18, 19). Due to these advantages, percutaneous kyphoplasty (PKP) has increasingly become the preferred surgical intervention for OVCF. Nevertheless, recent studies have highlighted several drawbacks of PKP. First, balloon dilation often results in uneven or localized cement accumulation within the vertebral body, which can lead to stress concentration and increase the risk of re-fracturing the treated vertebra or adjacent segments (20–23). Second, excessive attempts to restore vertebral height during balloon inflation may rupture the cortical bone, leading to severe complications such as delayed cement displacement or extrusion (24, 25). Moreover, the use of balloon-expandable instruments adds significantly to the overall treatment cost.

The comparative therapeutic PVP and PKP for OVCF remains debated, with the internal distribution of bone cement identified as a pivotal factor influencing long-term outcomes. Prior research suggests that a diffuse cement distribution with strong anchorage to trabecular bone is more effective in sustaining spinal alignment and reducing the risk of cement migration and extrusion. Current literature mainly relies on subjective, morphology-based grading systems to assess cement distribution, which lack reproducible quantification and fail to directly reflect biomechanical integration at the trabecular level. This study introduces a novel approach using 3D reconstruction and quantitative parameters for an objective evaluation of cement dispersion. This method allows for comparison of distribution patterns between PVP and PKP and establishes a direct, clinically relevant link between these parameters and key clinical outcomes. This study examines how bone cement distribution in PVP and PKP procedures affects vertebral height, Cobb angle correction, and symptom recurrence. The findings are clinically significant, guiding surgical technique choices and cement injection protocols to enhance long-term stability and reduce complications like adjacent fractures and correction loss.

## 2 Materials and methods

### 2.1 Selection of patients

This study protocol was approved by the Ethics Committee of our hospital (approval no.: KY2025284) and conducted in compliance with the Declaration of Helsinki. As this study is retrospective in nature and exclusively utilizes patients’ imaging data, with rigorous anonymization of identifiable patient information maintained throughout the research process, the requirement for obtaining patient informed consent was waived. In this study, we reviewed the clinical data of patients with OVCF admitted to our hospital during January 2020–2023. Patients were allocated to either the PVP or PKP group based on their autonomous choice after comprehensive preoperative counseling, which included detailed information regarding the procedures, associated medical costs, and anticipated insurance coverage. The inclusion criteria for the patients were as follows: (1) age ≥60 years or with a bone mineral density (BMD) of T ≤-2.5, as measured by dual-energy X-ray absorptiometry, (2) a known history of hypochondriac and/or back pain, without or with limited mobility, (3) demonstrating acute, single OVCF as confirmed by magnetic resonance imaging, (4) availability of complete postoperative follow-up CT, and (5) a follow-up record of a minimum of 24 months after the surgery, with the most recent X-ray examination conducted at least 24 months after the surgery. The exclusion criteria for the patients were as follows: (1) fractures caused by pathological factors, such as primary or secondary tumors and vertebral infections; (2) vertebral fractures resulting in spinal cord compression; (3) space-occupying lesions in the vertebrae; (4) multiple vertebral fractures; (5) a history of spinal surgery.

### 2.2 Statistical methods for addressing confounding

Given the non-randomized design of this study, we addressed potential confounding using two methods. First, we performed 1:1 propensity score matching (PSM) using the MatchIt package in R. This was conducted with the nearest neighbor method and a caliper width of 0.1 standard deviations of the logit of the propensity score. The covariates included in the propensity score model were Age, Gender, BMD, Duration, and T/L spine ratio. Second, as a sensitivity analysis, we used Inverse Probability of Treatment Weighting (IPTW) with the WeightIt package, using the same set of covariates. Covariate balance before and after matching or weighting was assessed using the Standardized Mean Difference (SMD). A good balance was considered to be achieved when the absolute SMD for all covariates was less than 0.1. All the aforementioned analyses were repeated on the matched and weighted datasets. All statistical analyses and visualizations were performed using R software (version 4.4.1). A two-sided P-value < 0.05 was considered statistically significant.

### 2.3 Surgical approaches

**Figure 1:**
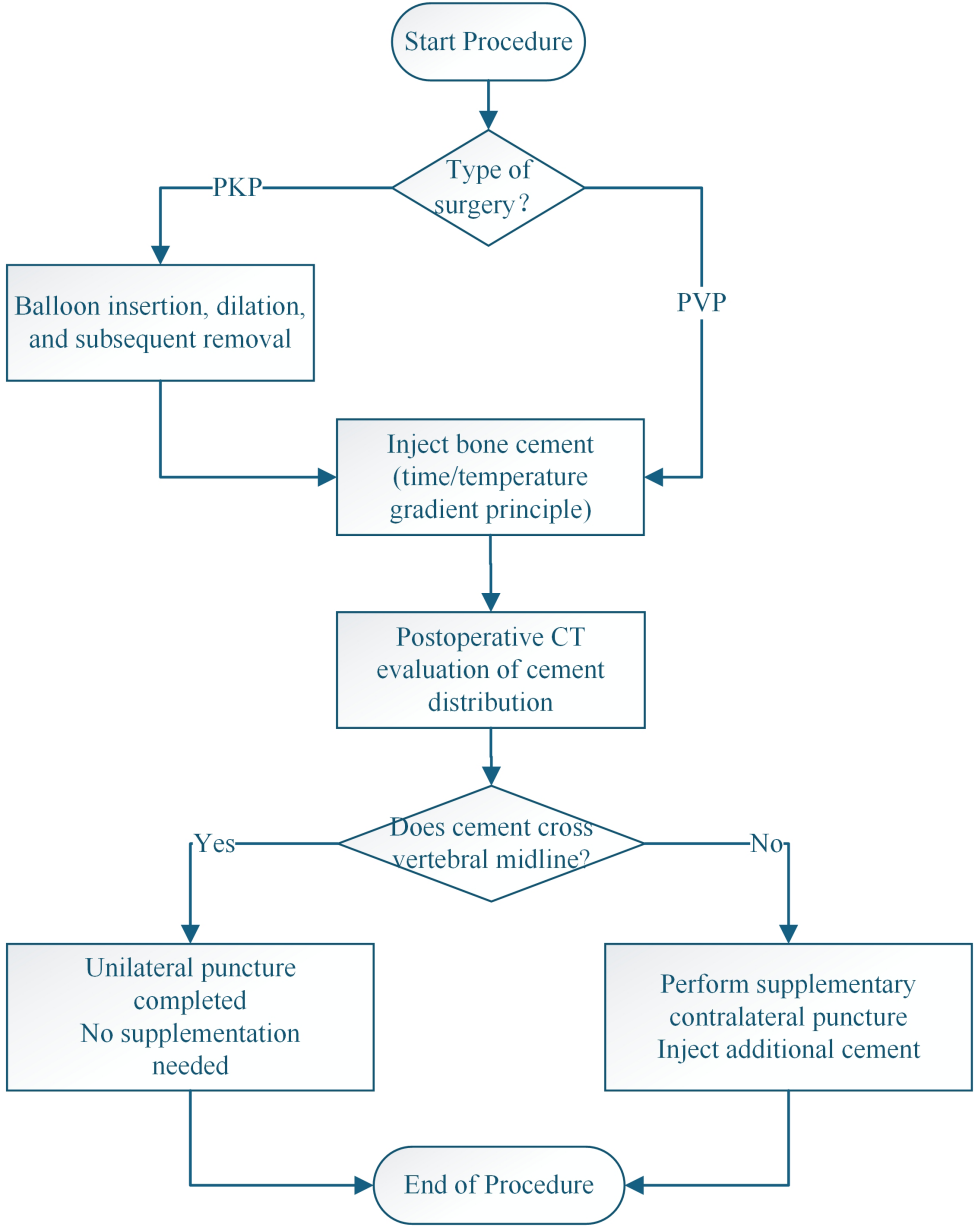
Flowchart for decision-making on unilateral puncture and supplementary contralateral Puncture during percutaneous vertebroplasty and kyphoplasty.

All PVP and PKP procedures were conducted by two spinal surgeons (i.e., W.Q. and X.S.), each of whom had received standard training in PVP and PKP and had >10 years of experience with these procedures. All patients were treated via a unilateral puncture technique under local anesthesia. The fractured vertebra was localized by C-arm fluoroscopy, and the entry point was marked at the midpoint of the transverse process, 3–5-mm lateral to the outer edge of the pedicle projection. Successful puncture was confirmed when, on an anteroposterior (AP) view, the puncture needle extended to the inner edge of the pedicle, whereas, on the lateral view, the needle tip was positioned at the posterior margin of the vertebral body. If the needle extended 5-mm anterior to the posterior margin of the vertebral body, a guidewire was implanted. With reference to the lateral view, guidewires positioned in the anterior one-third of the vertebral body that reached or exceeded the midline of the spinous process on the AP view were then exchanged for a bone cement injection catheter. Next, a 3-mm drill was inserted through the cannula, and its position was confirmed to be located at the anterior one-third of the vertebral body, as observed under lateral fluoroscopy. After the drill removal, in the PKP group, balloons were implanted in the patients, which were then expanded using a 200-Pa pressure. After removing the balloon, bone cement was injected. In the PVP group, bone cement was directly injected after the drill removal and incrementally injected using time- and temperature-gradient principles. The cement-injection process was monitored using C-arm lateral fluoroscopy. After delivering the cement, the plunger was promptly separated, and the working cannula was rotated. Once the cement had hardened, the cannula was carefully withdrawn. If postoperative CT imaging (reviewed in both anteroposterior and lateral planes) confirmed that the cement distribution did not extend beyond the vertebral midline, indicating inadequate dispersion from the initial unilateral puncture, a supplementary contralateral pedicle puncture was performed to administer additional cement (**Figure 1**) .

All patients received back braces for a period of 1–2 months after the procedure and were treated with osteoporotic medications after the surgery.

### 2.4 Evaluation of clinical parameters

The clinical parameters assessed in this study included a comparative analysis of the baseline characteristics such as gender, age, disease duration, and BMD. The intraoperative parameters analyzed included surgery duration, intraoperative blood loss, frequency of C-arm fluoroscopy usage, and bone cement volume. The clinical efficacy of the two surgical procedures was evaluated based on the changes in VAS and ODI determined before the surgery, 1-day after the surgery, at 6 months postoperatively, and at the final follow-up examination.

### 2.5 Evaluation of radiological parameters

Anterior vertebral height (AVH) and local Cobb angle (CA) were determined as described by Lee et al. (26) and Kuklo et al. (27), respectively, and recorded at the same three time points as mentioned earlier.

The correction degree of CA = (preoperative CA - postoperative CA)/preoperative CA. The loss rate of the correction degree of CA = the correction degree of CA at 6 months or 2 years postoperatively - the correction degree of CA on the first day postoperatively. The improvement rate of AVH = (AVH on the first day postoperatively - preoperative AVH)/preoperative AVH. The loss rate of the improvement rate of AVH was calculated as the improvement rate of AVH at 6 months or 2 years postoperatively - the improvement rate of AVH on the first day postoperatively.

### 2.6 Evaluation of cement distribution

Postoperative CT data were exported in the DICOM file format and imported into Mimics 21.0 software. The targeted vertebral body was selected using the CT Bone tool. The Mask Threshold function was applied to adjust the grayscale values of the bone tissue within the range of 226–3071 Hu, and the Edit Mask function was used to remove vertebral appendages while preserving the vertebral body. Subsequently, the Smart Fill, Cavity Fill, or Edit Masks functions were employed to fill any gaps within the vertebral body. The 3D Calculate Part function was applied to reconstruct the three-dimensional model of the vertebral body. Finally, the Properties function was selected to record the vertebral body volume (VBV), and the previously reconstructed three-dimensional vertebral body model was deleted. The Threshold function was then adjusted to the grayscale values of bone cement, ranging 1000–3000 Hu. The Region Grow tool was used for the segmentation to reconstruct the bone cement model. The Properties function was utilized to calculate the cement surface area (CSA) and cement dispersion volume (CDV). The specific surface area (SSA) of the bone cement was calculated as SSA = CSA/CDV, and the volume fraction percent (VF%) was calculated as VF% = CDV/VBV. SSA and VF% were then applied to represent the dispersion degree of the bone cement (**Figure 1**). In addition, postoperative X-ray films were reviewed to analyze whether the bone cement distribution exceeded the midline of the vertebral body. The 12-point scoring method, as proposed by Liu et al. (28) was employed to compare the bone cement dispersion between the two groups. The correlation was calculated between the bone cement distribution pattern and the rate of deformity correction loss as well as the degree of clinical symptom improvement in the PKP group.

All investigators received identical training sessions to identify the critical parameters within the images, thereby standardizing the image analysis criteria in this study. These investigators then discussed the imaging data to reach a consensus regarding definitions for these images. After this, a unique image number was allocated to each patient and control to ensure blind evaluations regarding the demographics/groups of the patients. One orthopedic surgeon (i.e., Z.S.) evaluated images twice to assess their intra-observer agreement. To assess the interobserver agreement, two investigators (i.e., Z.S and S.C.Y) evaluated 50 AVH, CA, improvement rate of CA, improvement rate of AVH, loss rate of improvement in CA, and the loss rate of improvement in AVH, CSA, CDV, SSA, or VF% scores of randomly selected patients.

### 2.7 Analysis of complications

The incidence of complications, including cement leakage, adjacent vertebral fractures, and re-fractures of the augmented vertebrae, was compared between the study groups.

### 2.8 Statistical analysis

All data were statistically analyzed using the SPSS 27.0 software program. Normally distributed measurement data were presented as the means ± standard deviations, whereas data failing to achieve a normal distribution were expressed as medians, along with their lower and upper quartile values. Categorical data were presented as the number and/or percent of cases. T-tests or Mann–Whitney U-tests were performed to compare the differences in age, BMD, operative time, blood loss, frequency of C-arm fluoroscopy usage, AVH, CA, AVHRR, CCA, CSA, CDV, VBV, SSA, and VF%. The chi-square test was performed to compare the gender ratio composition, the distribution of fracture sites, the proportion of cases wherein bone cement distribution exceeded the midline, the rate of supplemental contralateral puncture, incidence rate of bone cement leakage, and associated complications. Analysis of variance (ANOVA) with Bonferroni and Tamhane corrections was employed to compare the AVH and CA measured before the surgery, a day before the surgery, and at the final follow-up examination. Pairwise comparisons of the VAS and ODI scores were conducted by using the Wilcoxon signed-rank test, with a corrected significance level of α = 0.017 for bilateral tests. To evaluate the relationships between bone cement parameters (e.g., cement score, relative surface area) and clinical outcomes (e.g., VAS score improvement, ODI index, AVH loss rate, corrected CA loss rate), Pearson’s rank-correlation coefficient was applied. The level of statistical significance was defined as a p< 0.050.

## 3 Results

### 3.1 Baseline information

The average medical cost in the PKP group (8,042 ± 1,608 USD) was significantly higher than that in the PVP group (4,316 ± 863 USD), reflecting a significant difference (p < 0.05). No significant differences were observed between the PVP (N = 101) and PKP (N = 95) groups in other baseline characteristics, including age, sex, BMD, surgical duration, and fracture location (p > 0.05, **Table 1**).

**Table 1:** Comparison of preoperative general information between two groups of patients.

Parameter	PVP (n=101)	PKP (n=95)	Z/χ²/T	P
Gender			0.018	0.894
Male	31 (30.69%)	30 (31.58%)		
Female	70 (69.31%)	65 (68.42%)		
Age (years)	72.01 ± 6.22	71.55 ± 4.61	0.594	0.553
BMI (kg/m²)	21.31 ± 4.27	21.59 ± 5.50	-0.389	0.698
BMD (T-score)	-3.30 ± 0.59	-3.23 ± 0.48	-0.923	0.357
Disease Duration (days)	17.71 ± 2.56	17.43 ± 2.62	0.761	0.448
Fracture Site			1.591	0.451
T8-T12	37 (36.63%)	41 (43.16%)		
L1-L3	52 (51.49%)	47 (49.47%)		
L4-L5	12 (11.88%)	7 (7.37%)		
Medical Expenses (USD)	4316 ± 863	8042 ± 1608	-20.033	<0.001

### 3.2 Intraoperative parameters

The PKP group exhibited a significantly longer operative time (44.15 ± 10.09 min vs. 40.62 ± 11.71 min; p < 0.05) and required more frequent intraoperative C-arm fluoroscopy (24.26 ± 2.90 vs. 21.80 ± 3.35; p < 0.05) than the PVP group. The volume of bone cement used per vertebra was also higher in the PKP group (4.58 ± 0.46 mL vs. 4.12 ± 0.38 mL; p < 0.05). There was no significant difference in intraoperative blood loss between the groups (p > 0.05, **Table 2**).

**Table 2:** Comparison of surgical indicators between two groups of patients.

Parameter	PVP(n=101)	PKP(n=95)	Z/χ²/T	P
Surgical Time (min)	40.62 ± 11.71	44.15 ± 10.09	-2.371	0.019
Bone Cement Volume (mL)	4.12 ± 0.38	4.58 ± 0.46	-8.125	<0.001
Fluoroscopy Times (times)	21.80 ± 3.35	24.26 ± 2.90	-5.530	<0.001
Intraoperative Blood Loss (mL)	16.05 ± 1.55	15.67 ± 1.31	1.860	0.065

### 3.3 Clinical findings

Both groups demonstrated significant improvements in thoracolumbar pain, measured by VAS scores, and functional disability, assessed by ODI scores, from preoperative to postoperative evaluations at 1 day and during follow-up. However, at the 2-year follow-up, patients in the PKP group experienced a marked recurrence and worsening of thoracic back pain, with median VAS scores increasing from 2.00 [interquartile range (IQR): 2.00–3.00] to 3.00 (IQR: 2.00–5.00). Simultaneously, functional status deteriorated, with ODI scores rising from 21.60 (IQR: 20.20–23.00) to 28.80 (IQR: 24.10–40.50). At the 2-year follow-up, VAS and ODI scores in the PKP group were significantly higher than those in the PVP group (p < 0.05, **Table 3**), indicating persistent pain and functional decline.

**Table 3:** Comparison of VAS and ODI scores during follow-up between two groups of patients.

Parameter	PVP (n=101)	PKP (n=95)	Z/χ²/T	P
VAS Score				
Preoperative	8.00 (7.00,8.00)	8.00 (7.00,8.00)	-0.684	0.494
Postoperative Day 1	2.00 (1.00,2.00)	2.00 (1.00,2.00)	-0.807	0.420
Postoperative 6 Months	2.00 (1.00,2.00)	2.00 (2.00,3.00)	-0.504	0.075
Postoperative 2 Years	2.00 (2.00,3.00)	3.00 (2.00,5.00)	-2.052	0.014
ODI Index				
Preoperative	61.80 (60.15,64.25)	61.25 (60.40,63.70)	-0.895	0.371
Postoperative Day 1	22.00 (20.80,23.35)	21.60 (20.20,23.00)	-1.612	0.107
Postoperative 6 Months	22.40 (20.90,23.25)	23.60 (22.20,24.80)	-1.637	0.102
Postoperative 2 Years	21.50 (19.95,24.75)	28.80 (24.10,40.50)	-4.127	<0.001

### 3.4 Radiological findings

**Figure 2:**
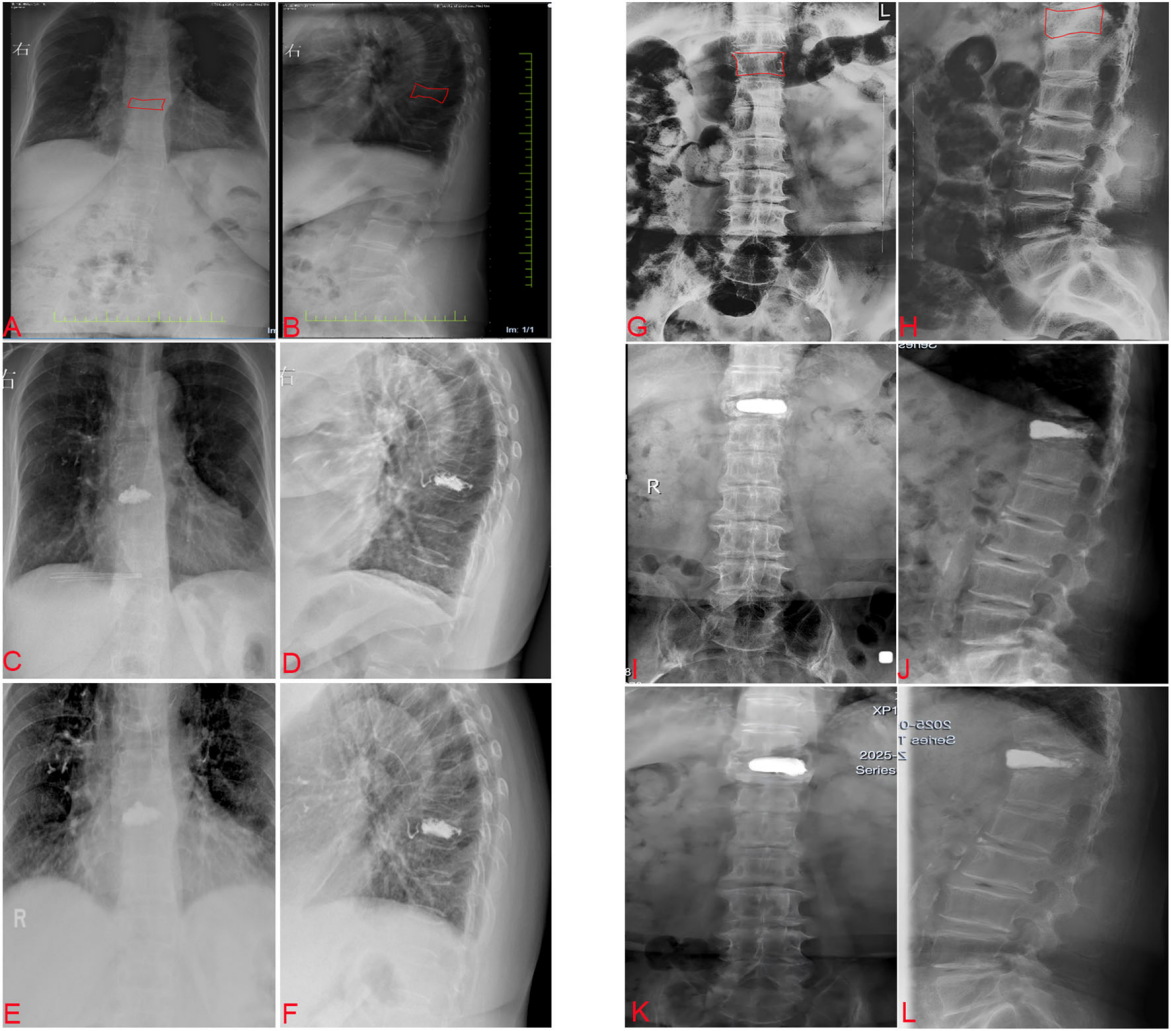
Comparison of typical cases between PVP and PKP. PVP **(A-F)** 67Y, F, T9 OVCF. **(A)** Preoperative anteroposterior X-ray view of the thoracolumbar spine. **(B)** Preoperative sagittal X-ray view of the thoracolumbar spine. **(C)** Anteroposterior X-ray view of the thoracolumbar spine at 6 months postoperatively. **(D)** Sagittal X-ray view of the thoracolumbar spine at 6 months postoperatively. **(E)** Anteroposterior X-ray view of the thoracolumbar spine at 2 years postoperatively. **(F)** Sagittal X-ray view of the thoracolumbar spine at 2 years postoperatively. PKP **(G-L)** 71Y, F, T11 OVCF. **(G)** Preoperative anteroposterior X-ray view of the thoracolumbar spine. **(H)** Preoperative sagittal X-ray view of the thoracolumbar spine. **(I)** Anteroposterior X-ray view of the thoracolumbar spine at 6 months postoperatively. **(J)** Sagittal X-ray view of the thoracolumbar spine at 6 months postoperatively. **(K)** Anteroposterior X-ray view of the thoracolumbar spine at 2 years postoperatively. **(L)** Sagittal X-ray view of the thoracolumbar spine at 2 years postoperatively. PVP, percutaneous vertebroplasty, PKP, percutaneous kyphoplasty, OVCF, osteoporotic vertebral compression fractures.

Both groups achieved significant postoperative improvements in AVH and local CA correction at all follow-up intervals. The PKP group exhibited superior anatomical restoration at 1 day, 6 months, and 2 years postoperatively in terms of AVH and local CA. Nonetheless, by the 2-year follow-up, both groups showed a degree of correction loss (**Figure 2**). The extent of correction loss was significantly greater in the PKP group, with local CA deterioration of 7.64% ± 1.12% compared with 1.10% ± 0.58% in the PVP group (p < 0.05), and AVH loss of 7.50% ± 0.97% versus 1.45% ± 0.57% (p < 0.05, **Table 4**). These results suggest that although PKP offers better initial structural correction, it is associated with greater long-term anatomical regression.

**Table 4:** Comparison of imaging data between two groups of patients.

Parameter	PVP(n=101)	PKP(n=95)	Z/χ²/T	P
Cobb Angle (°)				
Preoperative	15.06 ± 1.42	15.29 ± 1.31	1.362	0.175
Postoperative Day 1	12.04 ± 1.25	10.62 ± 0.99	2.630	0.007
Correction Degree (%)	21.00 ± 6.30	29.34 ± 7.23	-2.767	<0.001
Postoperative 6 Months	12.21 ± 1.25	11.80 ± 0.98	1.973	0.065
Correction Degree (%)	20.47 ± 6.23	26.05 ± 7.35	-1.470	<0.001
Correction Degree Loss (%)	0.53 ± 0.36	3.29 ± 0.41	-1.924	0.017
Postoperative 2 Years	12.65 ± 1.30	12.21 ± 1.24	-2.268	0.024
Correction Degree (%)	19.90 ± 7.74	21.70 ± 8.78	4.152	0.012
Correction Degree Loss (%)	1.1 ± 0.58	7.64 ± 1.12	-12.383	<0.001
AVH (mm)				
Preoperative	13.55 ± 1.26	13.64 ± 2.36	-0.342	0.731
Postoperative Day 1	15.99 ± 1.05	17.66 ± 2.06	-3.694	0.005
Restoration Rate (%)	19.83 ± 2.35	27.17 ± 2.86	-6.723	<0.001
Postoperative 6 Months	15.41 ± 1.08	16.80 ± 2.08	-3.641	<0.001
Restoration Rate (%)	19.12 ± 2.18	24.83 ± 2.54	-7.802	<0.001
Restoration Rate Loss (%)	0.71 ± 0.21	2.34 ± 0.28	-2.342	0.004
Postoperative 2 Years	15.09 ± 1.22	15.58 ± 2.33	0.489	0.034
Restoration Rate (%)	18.38 ± 2.56	19.67 ± 2.75	-3.015	0.003
Restoration Rate Loss (%)	1.45 ± 0.57	7.50 ± 0.97	-12.347	<0.001

### 3.5 Cement distribution

The rate of supplemental contralateral puncture did not differ significantly between the PVP (9.90%) and PKP (8.42%) groups (p > 0.05). According to Liu’s bone cement distribution scoring system, the PVP group achieved significantly better cement dispersion (median: 10.00, IQR: 9.00–10.00) than the PKP group (median: 8.00, IQR: 8.00–9.00), with statistical significance confirmed by the Mann–Whitney U test (p < 0.05). The PKP group demonstrated a significantly higher cement dispersion volume (CDV) compared to the PVP group (7.65 ± 0.58 cm³ vs. 6.85 ± 0.52 cm³, p < 0.05), although no significant differences were found between the groups in vertebral body volume (p > 0.05). Consistent with the CDV findings, the volume fraction percentage (VF%) was also significantly higher in the PKP group (30.81 ± 3.92% vs. 27.42 ± 2.85%, p < 0.05). Despite the greater cement volume used in PKP, the PVP group showed superior outcomes in CSA (40.18 ± 3.01 cm² vs. 31.96 ± 4.40 cm²) and SSA (5.66 ± 0.61 cm²/cm³ vs. 4.41 ± 0.67 cm²/cm³), with all differences reaching statistical significance (p < 0.05, **Table 5**).

**Table 5:** Comparison of bone cement parameters between two groups of patients.

Parameter	PVP(n=101)	PKP(n=95)	Z/χ²/T	P
Bone Cement Distribution Score	10.00(9.00,10.00)	8.00(8.00,9.00)	-9.038	<0.001
CSA (cm²)	40.18 ± 3.01	31.96 ± 4.40	15.167	<0.001
CDV (cm³)	6.85 ± 0.52	7.65 ± 0.58	-10.246	<0.001
SSA	5.66 ± 0.61	4.41 ± 0.67	13.656	<0.001
VBV(cm^3^)	24.98 ± 2.28	24.83 ± 2.26	0.463	0.644
VF%(%)	27.42 ± 2.85	30.81 ± 3.92	-7.893	<0.001
Supplemental Contralateral Puncture	10 (9.90%)	8 (8.42%)	0.141	0.707

**Figure 3:**
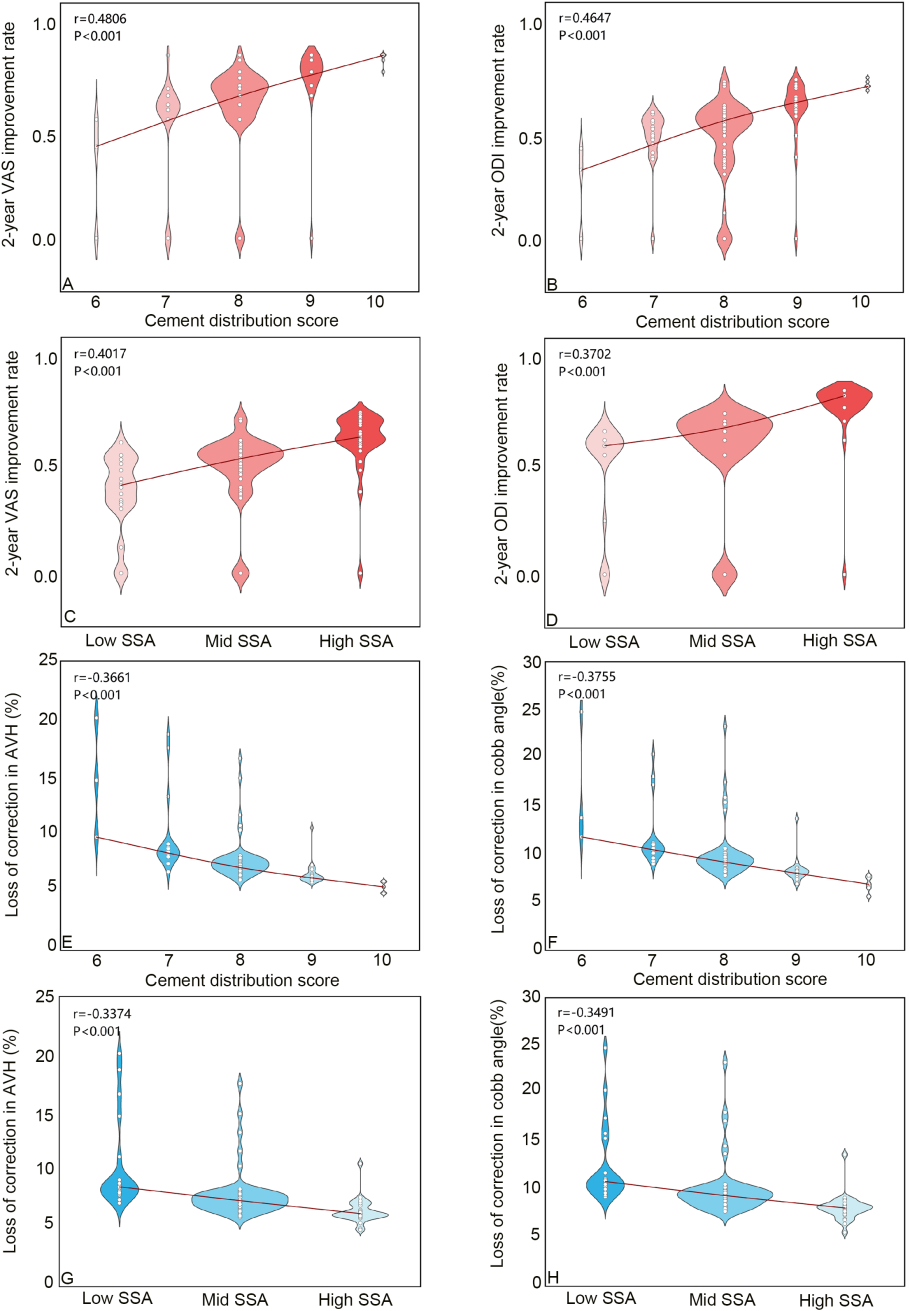
Correlation between bone cement distribution score and clinical symptom improvement as well as degree of deformity correction. **(A)** Correlation between cement distribution score and 2-year VAS improvement rate. **(B)** Correlation between cement distribution score and 2-year ODI improvenent rate. **(C)** Correlation between SSA and 2-year VAS improvement rate. **(D)** Correlation between SSA and 2-year ODI improvement rate. **(E)** Correlation between cement distribution score and loss of correction in AVH (%). **(F)** Correlation between cement distribution score and loss of correction in cobb angle (%). **(G)** Correlation between SSA and loss of correction in AVH (%). **(H)** Correlation between SSA and loss of correction in cobb angle (%). VAS, visual analogue scale, ODI, oswestry disability index, SSA, specific surface area, AVH, Anterior vertebral body height.

Correlation analysis revealed significant positive relationships between cement distribution scores, SSA, and the degree of clinical improvement in VAS scores and ODI indices. In contrast, these cement-related parameters were negatively correlated with AVH loss and CA correction loss, all showing significant associations (p < 0.05, **Figures 3**).

### 3.6 Complications

The incidence of bone cement leakage was significantly higher in the PVP group (19.8%) compared with the PKP group (8.42%) (p < 0.05). Adjacent vertebral fractures were significantly more common in the PKP group (9 cases) than in the PVP group (2 cases), with the difference reaching statistical significance (p < 0.05). Similarly, refracture of the treated vertebra occurred more frequently in the PKP group (8 cases) than in the PVP group (2 cases), and this difference was also significant (p < 0.05, **Table 6**).

**Table 6:** Comparison of postoperative complications between two groups of patients.

Parameter	PVP(n=101)	PKP(n=95)	Z/χ²/T	P
Bone Cement Leakage	20 (19.80%)	8 (8.42%)	5.128	0.024
Adjacent Vertebral Fracture	2 (1.98%)	9 (9.45%)	5.189	0.023
Refracture	2 (1.98%)	8 (8.42%)	4.194	0.041

## 4 Discussion

PVP and PKP surgeries involve the injection of bone cement into the vertebral body through minimal trauma to stabilize the fractured ends within the vertebra and reconstruct spinal stability, thereby providing immediate relief from chest or back pain in patients (7, 8). Initially, PVP was the preferred treatment for osteoporotic vertebral compression fractures (OVCF). Subsequently, Reiley et al. and Belkoff et al. introduced modifications to the PVP procedure by incorporating balloon kyphoplasty before bone cement injection (17). This approach effectively restored vertebral height and corrected spinal kyphosis. More importantly, numerous studies suggest that balloon expansion can effectively compress and seal fracture lines within the vertebra, reducing bone cement leakage (18, 19). Consequently, despite the significantly higher medical costs associated with PKP compared to PVP, PKP has become the primary surgical approach for treating OVCF.

The choice between unilateral and bilateral surgical approaches warrants consideration. In our institution, the unilateral puncture technique was primarily employed, with contralateral supplementation reserved for cases demonstrating inadequate cement dispersion across the vertebral midline on postoperative CT evaluation. This strategy aimed to minimize surgical trauma and radiation exposure while ensuring adequate cement distribution. The comparable rates of supplemental contralateral puncture in both groups (9.90% in PVP vs. 8.42% in PKP) suggest that most patients achieved satisfactory outcomes through the unilateral approach alone. The unilateral technique offers advantages including reduced operative time, decreased tissue damage, and lower radiation exposure. However, when cement distribution is suboptimal, supplemental contralateral puncture becomes necessary to enhance biomechanical stability and reduce the risk of correction loss.

With the global dissemination of PKP, many studies have found that although PKP achieves more pronounced short-term vertebral height restoration, the excessive aggregation of bone cement leads to increased stress, making patients more susceptible to re-fracture of the augmented vertebra or adjacent vertebral fractures postoperatively (20–23). Many studies posit that while the balloon used in PKP can expand the vertebral body and correct kyphosis, the cavity formed by balloon expansion confines bone cement distribution primarily within the expanded area, preventing effective interdigitation with the surrounding trabecular bone (24, 25). Furthermore, the stiffness of the solidified bone cement significantly exceeds that of the osteoporotic vertebra, thereby increasing the likelihood of re-fracture in the augmented vertebra or adjacent vertebrae following PKP.

Through retrospective analysis, this study found that both PKP and PVP surgeries significantly alleviated thoracic and back pain in patients in the short term. Compared to PVP, PKP demonstrated superior efficacy in restoring anterior vertebral height and improving local Cobb angle in the short term, consistent with findings from previous studies (14–16). However, during the follow-up period, this study observed a significant loss of anterior vertebral height restoration and Cobb angle correction in the PKP group. Concurrently, patients in the PKP group experienced recurrent exacerbation of thoracic and back pain and ODI dysfunction at the final follow-up. Further analysis of postoperative imaging data revealed that, according to Liu et al’s bone cement distribution score, the PVP group exhibited significantly better bone cement distribution than the PKP group. However, Liu et al’s method, like many others, evaluates bone cement distribution based on its morphology, which is highly subjective and exhibits poor interobserver agreement (29–32). Moreover, factors such as bone cement texture, temperature, viscosity, injection technique, and vertebral fracture morphology influence bone cement morphology within the vertebra, limiting the clinical value of evaluating bone cement distribution solely based on its shape. Subsequently, scholars proposed using the ratio of bone cement volume to vertebral volume to assess bone cement distribution (33, 34). However, due to the influence of bone cement’s fluid properties and the pressure within the cavity containing the bone cement, even when the same volume of bone cement is injected, its actual volume within the vertebra may vary. Most importantly, solely pursuing the volume of bone cement within the vertebra without considering its distribution pattern is unscientific.

To overcome the limitations of previous bone cement distribution evaluation methods, this study innovatively employed Mimics software to measure the surface area and volume of bone cement within the vertebra postoperatively, using the ratio of bone cement surface area to volume to evaluate bone cement distribution. This approach allows for the calculation of the surface area of bone cement in contact with vertebral bone per unit volume, providing a more intuitive and precise quantification of the contact area between bone cement and trabecular bone. Furthermore, this study analyzed the correlation between bone cement distribution and loss of correction in PKP patients using both Liu et al’s scoring method and the proposed approach. The findings revealed that a larger relative surface area of bone cement correlated with stronger ability to maintain correction. Compared to PKP patients, PVP patients exhibited better bone cement distribution patterns and larger contact areas between bone cement and trabecular bone, which may explain the absence of significant correction loss in PVP patients postoperatively.

The distribution pattern of bone cement within the vertebra not only influences deformity correction but also contributes significantly to re-fracture of the augmented vertebra and adjacent vertebral fractures (35–37). This study found that the incidence of re-fracture in the augmented vertebra and adjacent vertebral fractures was higher in the PKP group than in the PVP group. Previous literature suggests that after balloon kyphoplasty in PKP creates a cavity within the vertebra, bone cement tends to aggregate into clumps, failing to interdigitate with trabecular bone and even forming necrotic bone areas between the bone cement and vertebral bone (24, 25). Consequently, when the augmented vertebra is subjected to mechanical stress again, the highly rigid bone cement can easily cause fractures in the fragile vertebral bone. Additionally, the aggregated bone cement in PKP significantly increases the stiffness of the operated vertebra, particularly when larger volumes of bone cement are injected, leading to increased stress on adjacent vertebrae and a higher incidence of adjacent vertebral fractures. In contrast, in the PVP group, the extensive interaction area between bone cement and trabecular bone may disperse stress more effectively, potentially explaining the lower incidence of re-fracture in the augmented vertebra and adjacent vertebral fractures in this group. However, further confirmation through biomechanical experiments and finite element analysis is necessary to validate these findings.

The difference in long-term outcomes likely stems from variations in biological integration and mechanical load transfer at the “cement-bone interface” between the two methods. Ideally, cement augmentation should achieve maximum microscopic interlocking with the bone trabeculae. In PVP, injecting cement under moderate pressure enhances its penetration into trabecular spaces, creating strong microscopic mechanical interlocking, as supported by experimental models (38). This “infiltrative” distribution increases contact area and enhances bond strength through microscopic interlocking. In contrast, during PKP, balloon expansion compresses and disrupts the surrounding bone’s architecture, creating a smooth, dense cavity wall. Cement solidifies into an isolated mass, relying on macroscopic congruence rather than microscopic interlocking. Li et al. (24) noted that post-PKP, cement coalesces, hindering interdigitation and potentially causing bone necrosis at the interface, compromising integration (24). A poorly integrated interface can lead to micromotion and fatigue failure under long-term cyclic loading, affecting mechanical load transfer. Efficient load transfer through the augmented vertebra is crucial for handling spinal load. PVP’s diffuse cement network interweaves with trabeculae, allowing seamless stress transfer and preventing local stress concentration, thus protecting vertebral integrity and reducing stress on adjacent segments. In contrast, PKP forms a central cement mass with a higher elastic modulus than the surrounding bone, creating a “stress riser” that concentrates stress at the cement-bone interface and causes uneven load distribution on the superior endplate. This offers a clear mechanical explanation for why PKP is more prone to postoperative refractures and fractures in nearby vertebrae. Finite element analysis shows that uneven cement clumps cause abnormal load transfer and stress concentration in adjacent vertebrae (39). Moreover, biomechanical tests show that strengthening a vertebral body raises the risk of adjacent vertebrae failing (40). Frankel et al. (20) clinically demonstrated that PKP significantly increases the short-term risk of adjacent fractures compared to PVP (20).In essence, PVP’s cement distribution is superior due to its enhanced biological integration and mechanical behavior, leading to better interfacial interlocking and stress dispersion. This underpins its long-term stability and lower complication rates. Although PKP initially restores height more effectively, its interfacial properties may lead to long-term mechanical issues.

However, this study’s findings should be interpreted with caution due to several limitations. This single-center retrospective study, conducted by a consistent surgical team in one orthopedic department, ensures consistent techniques but limits generalizability. Differences in patient selection, surgical methods, materials, and rehabilitation across centers could affect outcomes. Future prospective multi-center studies with standardized protocols are needed for validation. Second, the study uses a non-randomized design, allowing patients to choose between PVP and PKP after thorough counseling. This respects patient autonomy and mirrors real-world decisions but introduces selection bias. Choices may be influenced by factors like socioeconomic status, insurance, preference for newer technology, or cost sensitivity. Wealthier patients might opt for the costlier PKP, differing in baseline characteristics from those choosing PVP. These unmeasured factors could partly explain the PKP group’s higher rates of re-fracture and adjacent vertebral fracture. Therefore, the comparisons of the long-term effectiveness of the two procedures should be seen as observational associations within a specific context, rather than definitive causal conclusions. While an innovative imaging method was employed to measure cement distribution, the assessment metrics could be improved. The study did not directly measure complex biomechanical factors like microscopic bond strength at the cement-trabeculae interface or detailed stress distribution within the cemented vertebra. These insights are essential for fully understanding the impact of cement distribution on vertebral biomechanics and long-term stability. In summary, recognizing these limitations enhances the study’s value by providing context. The findings offer key hypotheses and guide future research. Further studies should address these limitations with prospective, multi-center, randomized controlled trials and advanced biomechanical tools to clarify the long-term benefits of PVP and PKP in treating OVCF.

## 5 Conclusions

Both PVP and PKP are widely used in the management of OVCF and are capable of delivering favorable short-term clinical outcomes and anatomical correction. However, PKP involves a longer operative duration, greater radiation exposure, and higher treatment costs than PVP. Over the long term, due to the less optimal cement distribution achieved during PKP, patients in this group are more likely to experience a decline in deformity correction and may suffer recurrent thoracic or back pain symptoms. Therefore, PVP may be superior to PKP in maintaining long-term correction, particularly when bone cement distribution is optimized.

## Data Availability

The original contributions presented in the study are included in the article/supplementary material. Further inquiries can be directed to the corresponding author.

## References

[1] Edidin AA Ong KL Lau E Kurtz SM . Life expectancy following diagnosis of a vertebral compression fracture. Osteoporos Int. (2013) 24:451–8. doi: 10.1007/s00198-012-1965-2, PMID: 22422305

[2] Kado DM Duong T Stone KL Ensrud KE Nevitt MC Greendale GA . Incident vertebral fractures and mortality in older women: a prospective study. Osteoporos Int. (2003) 14:589–94. doi: 10.1007/s00198-003-1412-5, PMID: 12827222

[3] Borgstrom F Karlsson L Ortsater G Norton N Halbout P Cooper C . Fragility fractures in Europe: burden, management and opportunities. Arch Osteoporos. (2020) 15:59. doi: 10.1007/s11657-020-0706-y, PMID: 32306163 PMC7166207

[4] Johnell O Kanis JA . An estimate of the worldwide prevalence and disability associated with osteoporotic fractures. Osteoporos Int. (2006) 17:1726–33. doi: 10.1007/s00198-006-0172-4, PMID: 16983459

[5] Ensrud KE Thompson DE Cauley JA Nevitt MC Kado DM Hochberg MC . Prevalent vertebral deformities predict mortality and hospitalization in older women with low bone mass. Fracture Intervention Trial Research Group. J Am Geriatr Soc. (2000) 48:241–9. doi: 10.1111/j.1532-5415.2000.tb02641.x, PMID: 10733048

[6] Schlaich C Minne HW Bruckner T Wagner G Gebest HJ Grunze M . Reduced pulmonary function in patients with spinal osteoporotic fractures. Osteoporos Int. (1998) 8:261–7. doi: 10.1007/s001980050063, PMID: 9797911

[7] Hirsch JA Beall DP Chambers MR Andreshak TG Brook AL Bruel BM . Management of vertebral fragility fractures: a clinical care pathway developed by a multispecialty panel using the RAND/UCLA Appropriateness Method. Spine J. (2018) 18:2152–61. doi: 10.1016/j.spinee.2018.07.025, PMID: 30096377

[8] Parreira PCS Maher CG Megale RZ March L Ferreira ML . An overview of clinical guidelines for the management of vertebral compression fracture: a systematic review. Spine J. (2017) 17:1932–8. doi: 10.1016/j.spinee.2017.07.174, PMID: 28739478

[9] Alsoof D Anderson G McDonald CL Basques B Kuris E Daniels AH . Diagnosis and management of vertebral compression fracture. Am J Med. (2022) 135:815–21. doi: 10.1016/j.amjmed.2022.02.035, PMID: 35307360

[10] Hoyt D Urits I Orhurhu V Orhurhu MS Callan J Powell J . Current concepts in the management of vertebral compression fractures. Curr Pain Headache Rep. (2020) 24:16. doi: 10.1007/s11916-020-00849-9, PMID: 32198571

[11] Galibert P Deramond H Rosat P Le Gars D . Preliminary note on the treatment of vertebral angioma by percutaneous acrylic vertebroplasty. Neurochirurgie. (1987) 33:166–8., PMID: 3600949

[12] Barr JD Barr MS Lemley TJ McCann RM . Percutaneous vertebroplasty for pain relief and spinal stabilization. Spine (Phila Pa 1976). (2000) 25:923–8. doi: 10.1097/00007632-200004150-00005, PMID: 10767803

[13] Hiwatashi A Sidhu R Lee RK DeGuzman RR Piekut DT Westesson PL . Kyphoplasty versus vertebroplasty to increase vertebral body height: a cadaveric study. Radiology. (2005) 237:1115–9. doi: 10.1148/radiol.2373041654, PMID: 16304123

[14] Wang H Sribastav SS Ye F Yang C Wang J Liu H . Comparison of percutaneous vertebroplasty and balloon kyphoplasty for the treatment of single level vertebral compression fractures: A meta-analysis of the literature. Pain Physician. (2015) 18:209–22., PMID: 26000665

[15] Eck JC Nachtigall D Humphreys SC Hodges SD . Comparison of vertebroplasty and balloon kyphoplasty for treatment of vertebral compression fractures: a meta-analysis of the literature. Spine J. (2008) 8:488–97. doi: 10.1016/j.spinee.2007.04.004, PMID: 17588820

[16] Hulme PA Krebs J Ferguson SJ Berlemann U . Vertebroplasty and kyphoplasty: a systematic review of 69 clinical studies. Spine (Phila Pa 1976). (2006) 31:1983–2001. doi: 10.1097/01.brs.0000229254.89952.6b, PMID: 16924218

[17] Garfin SR Yuan HA Reiley MA . New technologies in spine: kyphoplasty and vertebroplasty for the treatment of painful osteoporotic compression fractures. Spine (Phila Pa 1976). (2001) 26:1511–5. doi: 10.1097/00007632-200107150-00002, PMID: 11462078

[18] Phillips FM Todd Wetzel F Lieberman I Campbell-Hupp M . An *in vivo* comparison of the potential for extravertebral cement leak after vertebroplasty and kyphoplasty. Spine (Phila Pa 1976). (2002) 27:2173–8. doi: 10.1097/00007632-200210010-00018, PMID: 12394934

[19] Yeom JS Kim WJ Choy WS Lee CK Chang BS Kang JW . Leakage of cement in percutaneous transpedicular vertebroplasty for painful osteoporotic compression fractures. J Bone Joint Surg Br. (2003) 85:83–9. doi: 10.1302/0301-620x.85b1.13026, PMID: 12585583

[20] Frankel BM Monroe T Wang C . Percutaneous vertebral augmentation: an elevation in adjacent-level fracture risk in kyphoplasty as compared with vertebroplasty. Spine J. (2007) 7:575–82. doi: 10.1016/j.spinee.2006.10.020, PMID: 17905320

[21] Fribourg D Tang C Sra P Delamarter R Bae H . Incidence of subsequent vertebral fracture after kyphoplasty. Spine (Phila Pa 1976). (2004) 29. doi: 10.1097/01.brs.0000142469.41565.2a, PMID: 15480139

[22] Harrop JS Prpa B Reinhardt MK Lieberman I . Primary and secondary osteoporosis’ incidence of subsequent vertebral compression fractures after kyphoplasty. Spine (Phila Pa 1976). (2004) 29:2120–5. doi: 10.1097/01.brs.0000141176.63158.8e, PMID: 15454702

[23] Majd ME Farley S Holt RT . Preliminary outcomes and efficacy of the first 360 consecutive kyphoplasties for the treatment of painful osteoporotic vertebral compression fractures. Spine J. (2005) 5:244–55. doi: 10.1016/j.spinee.2004.09.013, PMID: 15863078

[24] Li YX Guo DQ Zhang SC Liang D Yuan K Mo GY . Risk factor analysis for re-collapse of cemented vertebrae after percutaneous vertebroplasty (PVP) or percutaneous kyphoplasty (PKP). Int Orthop. (2018) 42:2131–9. doi: 10.1007/s00264-018-3838-6, PMID: 29464371

[25] Wei Q Zhan J Chen X Li H Guo W Liu Z . Development of a clinical predictive model for cement loosening after vertebral augmentation in osteoporotic vertebral compression fractures. BMC Musculoskelet Disord. (2024) 25:1052. doi: 10.1186/s12891-024-08111-8, PMID: 39702079 PMC11660886

[26] Lee ST Chen JF . Closed reduction vertebroplasty for the treatment of osteoporotic vertebral compression fractures. Technical note. J Neurosurg. (2004) 100:392–6. doi: 10.3171/spi.2004.100.4.0392, PMID: 15070152

[27] Kuklo TR Polly DW Owens BD Zeidman SM Chang AS Klemme WR . Measurement of thoracic and lumbar fracture kyphosis: evaluation of intraobserver, interobserver, and technique variability. Spine (Phila Pa 1976). (2001) 26. doi: 10.1097/00007632-200101010-00012, PMID: 11148647

[28] Liu J Tang J Liu H Gu Z Zhang Y Yu S . A novel and convenient method to evaluate bone cement distribution following percutaneous vertebral augmentation. Sci Rep. (2020) 10:16320. doi: 10.1038/s41598-020-73513-2, PMID: 33005025 PMC7530709

[29] Tanigawa N Komemushi A Kariya S Kojima H Shomura Y Omura N . Relationship between cement distribution pattern and new compression fracture after percutaneous vertebroplasty. AJR Am J Roentgenol. (2007) 189:W348–52. doi: 10.2214/AJR.07.2186, PMID: 18029848

[30] Wu W Zhang X Li X Yu S . The influence of diverse bone cement distribution patterns for metastatic vertebral lesions after bilateral percutaneous kyphoplasty. BMC Musculoskelet Disord. (2022) 23:713. doi: 10.1186/s12891-022-05680-4, PMID: 35883056 PMC9316733

[31] Yang K Zhu X Sun X Shi H Sun L Ding H . Bone cement distribution patterns in vertebral augmentation for osteoporotic vertebral compression fractures: a systematic review. J Orthop Surg Res. (2025) 20:568. doi: 10.1186/s13018-025-05868-z, PMID: 40468349 PMC12135570

[32] Zhou C Liao Y Huang S Li H Zhu Z Zheng L . Effect of cement distribution type on clinical outcome after percutaneous vertebroplasty for osteoporotic vertebral compression fractures in the aging population. Front Surg. (2022) 9:975832. doi: 10.3389/fsurg.2022.975832, PMID: 36034386 PMC9405186

[33] Fu Z Hu X Wu Y Zhou Z . Is there a dose-response relationship of cement volume with cement leakage and pain relief after vertebroplasty? Dose Resp. (2016) 14:1559325816682867. doi: 10.1177/1559325816682867, PMID: 28182178 PMC5283639

[34] Zhou C Liao Y Chen H Wang Y . Analysis of optimal volume fraction percentage and influencing factors of bone cement distribution in vertebroplasty using digital techniques. J Orthop Surg Res. (2023) 18:235. doi: 10.1186/s13018-023-03719-3, PMID: 36959652 PMC10035276

[35] Liang D Ye LQ Jiang XB Yang P Zhou GQ Yao ZS . Biomechanical effects of cement distribution in the fractured area on osteoporotic vertebral compression fractures: a three-dimensional finite element analysis. J Surg Res. (2015) 195:246–56. doi: 10.1016/j.jss.2014.12.053, PMID: 25634828

[36] Liebschner MA Rosenberg WS Keaveny TM . Effects of bone cement volume and distribution on vertebral stiffness after vertebroplasty. Spine (Phila Pa 1976). (2001) 26:1547–54. doi: 10.1097/00007632-200107150-00009, PMID: 11462084

[37] Tang J Wang S Wang J Wang X Li T Cheng L . Risk factors for secondary vertebral compression fracture after percutaneous vertebral augmentation: a single-centre retrospective study. J Orthop Surg Res. (2024) 19:797. doi: 10.1186/s13018-024-05290-x, PMID: 39593155 PMC11600641

[38] Baroud G Crookshank M Bohner M . High-viscosity cement significantly enhances uniformity of cement filling in vertebroplasty: an experimental model and study on cement leakage. Spine (Phila Pa 1976). (2006) 31:2562–8. doi: 10.1097/01.brs.0000240695.58651.62, PMID: 17047545

[39] Polikeit A Nolte LP Ferguson SJ . The effect of cement augmentation on the load transfer in an osteoporotic functional spinal unit: finite-element analysis. Spine (Phila Pa 1976). (2003) 28:991–6. doi: 10.1097/01.BRS.0000061987.71624.17, PMID: 12768136

[40] Berlemann U Ferguson SJ Nolte LP Heini PF . Adjacent vertebral failure after vertebroplasty. A biomechanical investigation. J Bone Joint Surg Br. (2002) 84:748–52. doi: 10.1302/0301-620x.84b5.11841, PMID: 12188498

